# The SWI/SNF ATP-dependent chromatin remodeling complex in cell lineage priming and early development

**DOI:** 10.1042/BST20230416

**Published:** 2024-04-04

**Authors:** Dhurjhoti Saha, Srinivas Animireddy, Blaine Bartholomew

**Affiliations:** Department of Epigenetics and Molecular Carcinogenesis, Center for Cancer Epigenetics, University of Texas MD Anderson Cancer Center, Houston, TX 77054, U.S.A.

**Keywords:** AT-hook, BAF, cell fate determination, embryonic stem cells, pluripotency, SWI/SNF

## Abstract

ATP dependent chromatin remodelers have pivotal roles in transcription, DNA replication and repair, and maintaining genome integrity. SWI/SNF remodelers were first discovered in yeast genetic screens for factors involved in mating type switching or for using alternative energy sources therefore termed SWI/SNF complex (short for SWItch/Sucrose NonFermentable). The SWI/SNF complexes utilize energy from ATP hydrolysis to disrupt histone-DNA interactions and shift, eject, or reposition nucleosomes making the underlying DNA more accessible to specific transcription factors and other regulatory proteins. In development, SWI/SNF orchestrates the precise activation and repression of genes at different stages, safe guards the formation of specific cell lineages and tissues. Dysregulation of SWI/SNF have been implicated in diseases such as cancer, where they can drive uncontrolled cell proliferation and tumor metastasis. Additionally, SWI/SNF defects are associated with neurodevelopmental disorders, leading to disruption of neural development and function. This review offers insights into recent developments regarding the roles of the SWI/SNF complex in pluripotency and cell lineage primining and the approaches that have helped delineate its importance. Understanding these molecular mechanisms is crucial for unraveling the intricate processes governing embryonic stem cell biology and developmental transitions and may potentially apply to human diseases linked to mutations in the SWI/SNF complex.

The development of a multicellular organism initiates with a single-celled zygote, which undergoes rapid cell division, giving rise to the formation of the inner cell mass (ICM). Embryonic stem cells (ESCs) isolated from this early stage are referred to as being in a naïve pluripotent state [1]. These cells possess the remarkable ability for self-renewal and can differentiate into various cell types in response to appropriate stimuli. The naïve pluripotent state is characterized by the expression of specific transcription factors (TFs), a hypomethylated genome, and is prominent during the pre-implantation stage of mouse development [2–4]. Following implantation, ESCs experience significant epigenetic changes leading to a global shift in gene expression. This transformation is accompanied by increased DNA methylation, a loss of self-renewal capacity, and a transition towards epithelialization [2,5,6]. This altered cellular state is recognized as the primed or epiblast cell state[1]. During this stage, cells express lineage-specific TFs and become primed to differentiate into the three germ layers, subsequently giving rise to various tissues and organs [7–9].

## Cell models for studying pluripotency and early mammalian development

The pluripotent state is highly transient during embryonic development, lasting only for a short period *in vivo*; however, cells representative of the pluripotent state can be captured and stably maintained *in vitro*. Mammalian ESCs were first cultured ex vivo from cells derived from the ICM of the blastocyst with the capacity to differentiate into any cell type. At first it was thought there was a single pluripotent state, but it now appears there is a continuum of different pluripotent states [10]. Early on the human and mouse ESCs were found to behave differently with mouse ESCs being more representative of cells in the pre-implantation stage and human ESCs representative of epiblast cells from post-implantation [11]. Later studies showed that human ESCs could be made that were more like mouse ESCs and these are collectively referred to as naïve stem cells [12]. Similarly, mouse ESCs can be cultured to resemble human ESCs and are referred to as EpiSC (epiblast stem cells) or primed stem cells [13,14]. For the purpose of this review we will refer to EpiSC simply as primed stem cells. The naïve and primed stem cells are representative of initial and final stages of pluripotency. Naïve and primed cell types resemble their counterparts in the embryo and featured by several distinct epigenetic, transcriptional, metabolic, and cell signaling changes (see Table 1). Although, these two cell types have limited representatives of embryogenesis, nonetheless proven to be invaluable in studies of early mammalian development.

**Table 1.** Comparision of various key features in during naïve to primed transition in mouse and human ESCs and early embryonic stages.

Key features	Naive pluripotent state				Primed pluripotent state				References
	Mouse		Human		Mouse		Human		
	*In vitro*	*In vivo*	*In vitro*	*In vivo*	*In vitro*	*In vivo*	*In vitro*	*In vivo*	
**Epigenetic regulation**									
Global DNA methylation	Hypo	Hypo	Hypo	Hypo	Hyper	Hyper	Hyper	Hyper	[15–20]
X chromosome inactivation in female cells	No	No	No	No	Yes	Yes	Yes	Yes	[21–24]
H3K27me3 on developmental genes	Low	Low	Low	Low	High	High	High	High	[25–29]
OCT4 enhancer switching	Distal	Distal	Distal	Distal	Proximal	Proximal	Proximal		[30–33]
Chromatin accessibility	High		High	High	Low		Low		[34–37]
**Transcriptional regulation**									
Priming markers (ZIC2, OTX2)	Low	Low	Low	Low	High	High	High	High	[2,26,30,38,39]
Pluripotency markers (KLFs, NANOG,)	High	High	High	High	Low	Low	Low	Low	[2,9,38,40–44]
ESRRβ	High	High	High	low	Low	Low	Low	Low	[43,45–48]
Cadherin	E- type	E- type	E- type	E-type	N- type	N- type	E-type		[26,49–52]
c-KIT expression	Yes	Yes	Yes	Yes	No	No	Yes	No	[53–57]
CD24/MHC class 1	Low	Low	Low	Low	High	High	High	High	[41,58,59]
**Metabolism**									
Oxidative phosphorylation	High	High	High		Low	Low	Low		[60–63]
Glycolysis	Low	Low	Low	Low	High	High	High	High	[61,64–67]
Lipid metabolism	High	High	High		Low	Low	Low		[61,68–70]
Polyamine metabolic pathway	High	High	High		Low	Low	Low		[71–73]
Threonine metabolism	High	High	High		Low	Low	Low		[73–75]
SAM levels	High	High	High		Low	Low	Low		[76,77]
α-keto glutarate	High	High			Low	Low			[78]
Amino acid/ATP	High	High	High		Low	Low	Low		[61,73]
**Cell signaling and cell cycle**									
MEK–ERK dependence	No	No	No		Yes	Yes	Yes		[2,26,79,80]
Wnt dependence	Yes	Yes	Yes	Yes	No	No	No		[2,81–83]
G-protein coupled signaling	High		High		Low		Low		[61,84,85]
Cell cycle	High		High		Low		Low		[61,86]
CDK2 dependency	Yes		Yes	No	No		No		[87,88]
Telomere length maintenance	Yes				No				[89]
DNA repair capacity	High				Low				[89]

There is a third class of stem cells representative of an intermediate state in pluripotency referred to as formative stem cells that have germline induction, a feature missing in both the naïve and primed stages. One type of cells with features of this type are called epiblast-like cells, which resemble cells in the pre-implantation epiblast that are maintained with bFGF and activin but unfortunately are heterogeneous [90,91]. On going efforts have uncovered better culture conditions for isolation of formative pluripotent stem cells whose transcription profile resembles that seen in pre-gastrulation formative epiblast and have super-bivalency at a large number of developmental genes [91–93]. The discovery of formative pluripotent cells indicates that the pluripotent state needs to be disassembled to some extent before lineage priming can be initiated.

## Metabolism in the different stages of pluripotency

Stem cells, owing to their rapid division, experience a heightened demand for metabolic precursors essential for DNA replication. Simultaneously, these cells necessitate substantial ATP hydrolysis, a process that significantly influences both the epigenetic and transcriptional landscape. There are major differences in the metabolic states of naïve and primed stem cells (Table 1) with naïve cells using both glycolysis and oxidative phosphorylation for energy and primed cells primarily glycolysis [10]. Another difference is fatty acid oxidation being required for naïve stem cell maintenance and not for the primed stage [60,64,94]. The intermediate formative stage between the naïve and primed stages has a strong reduction in mitochondrial respiration and increased levels of lipid metabolic enzymes compared with the naïve and primed stage [68,93,95].

**Figure 1.**
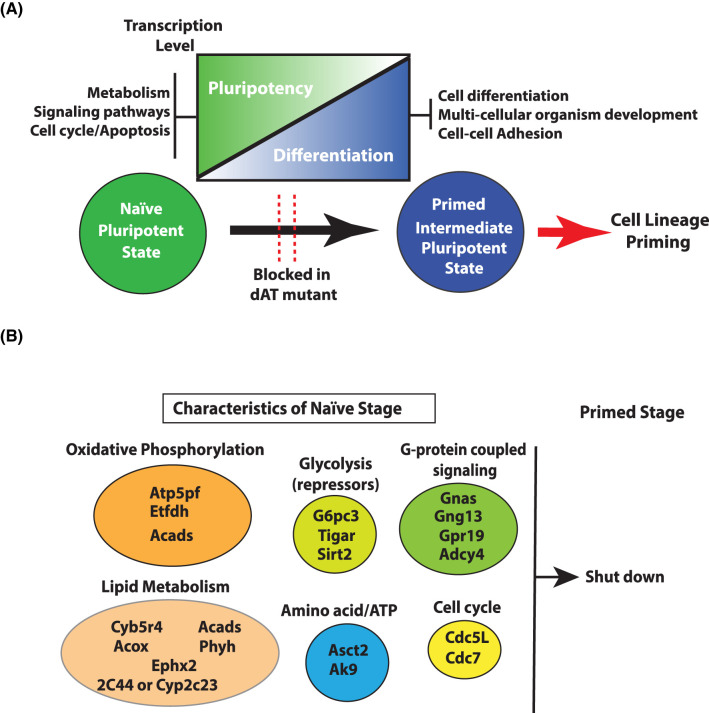
Key changes in major signaling pathways during naïve to primed stage transition. (**A**) Key changes in pathways in naïve to primed transition stage. Deletion/mutation of Brg1 AT-hook blocks these signaling pathways and affects cell lineage priming. (**B**) List of active metabolic genes/pathways in naïve pluripotent stage which are shoutdown in the primed stage. (Atp5pf = ATP synthase-coupling factor 6; Etfdh = electron transfer flavoprotein-ubiquinone oxidoreductase; Acads = short-chain specific acyl-CoA dehydrogenase; G6pc3 = glucose-6-phosphatase 3; Tigar = fructose-2,6-bisphosphatase; Sirt2 = NAD-dependent protein deacetylase sirtuin-2; Gnas = guanine nucleotide-binding protein G(s) subunit α isoform; Gng13 = guanine nucleotide-binding protein G(I)/G(S)/G(O) subunit γ-13; Gpr19 = G-protein coupled receptor 19; Adcy4 = adenylate cyclase type 4; Cyb5r4 = cytochrome b5 reductase 4; 2C44 or Cyp2c23 = cytochrome P450; Acox = peroxisomal acyl-coenzyme A oxidase 1; Ephx2 = bifunctional epoxide hydrolase 2; Phyh = phytanoyl-CoA dioxygenase, peroxisomal; Cdc5L = cell division cycle 5-like protein; Cdc7 = cell division cycle 7-related protein kinase; Asct2 = alanine, serine, cysteine transporter; Ak9 = protein adenylate kinase 9).

Some of the metabolic and signaling proteins involved in the transition from naïve to primed states are identified by tracking changes in nascent RNA transcription using techniques like PRO-seq [61]. These proteins are involved in (1) glycolysis, (2) oxidative phosphorylation, (3) lipid metabolism, (4) glutamine transport, (5) regulation of adenine nucleotide levels and (6) G-protein coupled signaling (Figure 1 and Table 1). The Sirt2, Tigar (fructose-2,6-bisphosphatase) and G6pc3 (glucose 6 phosphotase 3) genes are expressed primarily in mouse naïve ESCs and facilitate in down-regulating glycolysis. Sirt2 is an NAD-dependent deacetylase that deacetylates several glycolytic enzymes (GAPDH, PGK1, ENO1, PKM and ALDOA) and thereby inactivates them while also activating oxidative phosphorylation [96,97]. The genes encoding several mitochondrial proteins involved in oxidative phosphorylation and those in lipid metabolism are repressed in the primed stage, consistent with the switch to primarily glycolysis in the primed stage and the importance of lipids in pluripotency (see Figure 1). There is an increased need for glutamine due to rapid proliferation and is likely the reason for the increased expression of *Asct2* (alanine, serine, cysteine transporter 2) in the naïve compared with primed stage. Asct2 is a neutral amino acid transporter, whose preferred substrate is the conditionally essential glutamine [98]. Next, the *Ak9* gene encoding nucleoside-diphosphate kinase is expressed higher in naïve than primed cells and regulates the level of adenine nucleotides in line with the higher demand for ATP in the naïve stage [99]. The G-protein coupled signaling pathway is important for ESC proliferation and its physical properties and expression of several genes encoding various isoforms of the guanine nucleotide binding protein/receptor are more highly expressed in naïve than primed cells[100,101].

These metabolic and signaling changes that occur between the naïve and primed stages are conserved across mouse and human as well as in their pre and post implantation embryonic states reflect the reconfiguring of a pluripotent state in the transition to cell lineage priming and cell fate determination by removing basic inherent properties of pluripotency (Table 1).

## Molecular and epigenetic characteristics of the naïve and primed stages

Some of the characteristics that distinguish naïve from primed stem cells are the lack of female X-chromosome inactivation, the ability of self-renewal when MEK signaling is inhibited and to generate chimeric embryos [10]. Wnt/β-catenin signaling is needed for naïve stem cell self-renewal and inhibits the transition into primed stem cells [102,103]. Mouse naive ESCs divide unusually rapidly with a particular short G1-phase and ∼50–70% of the cell cycle is in the S-phase [104]. The G1 phase is shorter due to the unregulated or constitutive expression of the CDK2 (cyclin dependent kinase 2). The G1 phase becomes longer as cells begin to differentiate and synthesis of CDK2 become more tightly regulated and expressed at lower levels.

The metabolic changes described earlier are often tied to changes in the epigenetic state of naïve and primed stages. Typically, during the transition from the naïve to primed stage, genomic DNA undergoes a shift from hypomethylation to methylation, with trimethylation occurring at histone H3 lysine 27 in lineage regulatory genes during the primed stage [25,26,105]. These changes in DNA methylation parallel the observations in early embryogenesis, where DNA experiences global demethylation followed by remethylation. However, both pre- and post-implantation mouse epiblast cells maintain a hypomethylated state [106]. In the naïve stage, DNA demethylation at pluripotency genes is orchestrated by PRDM14 (PR domain containing 14), recruiting TET1/TET2 and repressing de novo methyltransferase [107]. Overexpression of PRDM14 in the primed stage induces a conversion to the naïve stage, facilitating OCT3/4 recruitment to the enhancers of naïve pluripotency genes [108]. Throughout this conversion, PRDM14 also represses primed stage-specific genes through PRC2 complex recruitment and accompanying trimethylation of lysine 27 of H3 (H3K27me3) [109]. In murine pre-implantation embryos, H3K27me3 and H3K4me3 possess distinct dynamics where H3K4me3, especially on promoter regions, occurs much more rapidly than that of H3K27me3 following fertilization, which is consistent with the major wave of zygotic genome activation at the two-cell stage [27]. Chromatin in the naïve stage adopts a more open state, enhancing transcription compared with the more closed state observed in the primed stage, akin to the patterns observed in pre- and post-implantation embryos[110].

The transition from naïve to primed stages involves substantial enhancer or cis-regulatory element switching. This is evident through changes in the localization of monomethylated lysine 4 of histone H3 (H3K4me1) and acetylated lysine 27 of histone H3 (H3K27ac), as well as the binding of TFs. Enhancers are either decommissioned or activated in a stage-specific manner [30,61,111]. Corresponding alterations in enhancer-promoter interactions are observed between the naïve and primed stages in human and mouse ESCs [112,113]. During this transition, enhancer-promoter interactions for the same gene are modified, as exemplified by OCT4. The OCT4 gene possesses distal and proximal enhancers, with the distal enhancer primarily responsible for activation in the naïve stage and the proximal enhancer assuming dominance in the primed stage [30,31]. These enhancers appear to be regulated by repressive histone modifications and DNA methylation in a stage-specific manner[32]. While pluripotency TFs remain crucial in both stages, they operate in different contexts. Oct4 TF, for instance, is essential for both pluripotency maintenance and exit, with its genomic localization undergoing global alterations in the transition from naïve to primed stages. The binding of Oct4 and Sox2, inferred by ATAC-seq, can be categorized into three classes: naïve or primed stage-specific and those present in both stages [30,61].

Despite the extensive work in this area, there is still much that is unknown about how changes in the transition to cell lineage priming are regulated and particularly the role of ATP-dependent chromatin remodeling in this process.

## Diversity of mammalian SWI/SNF complexes

A critical player in cell fate determination are the family of SWI/SNF ATP-dependent remodelers which are often referred to as BAF (BRG1 or BRM associated factors) complexes. At the heart of the SWI/SNF is an ATP-dependent DNA translocase that binds and translocates along the DNA wrapped around the nucleosomes, the most basic component of chromatin. DNA translocation along nucleosomal DNA can have one of three basic outcomes. The first is moving the nucleosome to different positions on DNA with the ability to create regions of free DNA devoid of bound histone [114]. Instead of merely rearranging nucleosome positions, SWI/SNF also has the capacity of completely displacing parts of nucleosome (a.k.a. H2A-H2B dimers) or entirely evicting nucleosomes from DNA [115,116]. A third property of ATP-dependent chromatin remodelers not observed with SWI/SNF complexes are the ability to change nucleosome composition by exchanging dimers with different histone isoforms (a.k.a. H2A-H2B for H2A.Z-H2B) [117].

There are several ways in which SWI/SNF is recruited to the correct target sites involving factors that together with SWI/SNF reciprocally facilitate each other's binding. DNA sequence-specific TFs like hormone receptors or pioneer TFs have been shown to recruit SWI/SNF [118–120]. The other mode of stabilizing SWI/SNF binding to genomic sites is through its interactions with post-translationally modified histones [121–123]. The 2-megadalton SWI/SNF complex has a wide arrange of various histone reader domains including bromodomain in Brg1, plant homology domain in BAF45 and chromodomain in BAF155 and BAF170, that can bind to variety of covalently modified residues residing in nucleosomes and increase the residence time of SWI/SNF on chromatin[124,125]. Transcriptional co-activators like p300/CBP, a histone acetyltransferase, and MLL3/4, a histone methyltransferase, have also been found to promote SWI/SNF binding to chromatin independent of their associated modification that is likely mediated through direct protein-protein interactions between the complexes [126–128]. There are likely other factors involved in SWI/SNF recruitment that have not yet been uncovered. In mammals, SWI/SNF is recruited to both promoter and enhancer regions to activate transcription and is highly enriched at super-enhancers, cis-regulatory regions known to determine cell type specificity [129].

Mammalian SWI/SNF has two catalytic subunits called BRG1 (SMARCA4) and BRM (SMARCA2) that contain the DNA-dependent ATPase domain and are assembled mutually exclusive of each other in distinct SWI/SNF complexes. There are 9–11 other subunits that assemble with the catalytic subunit to form a complete complex. These accessory subunits are encoded by 27 different genes and accounts for the diverse complex composition of SWI/SNF that varies in a cell-type specific manner [130]. There are three basic families of mammalian SWI/SNF called cBAF, PBAF and ncBAF/GBAF that differ by the composition of the accessory subunits and contain BRG1 or BRM. The PBAF (Polybromo-associated BAF complex) is distinguished from the cBAF (canonical BAF complex) by the incorporation of BAF180 (PBRM1) and BAF200 (ARID2) subunits and in cBAF BAF200 is replaced by BAF250A/B (ARID1A/B). Furthermore, PBAF lacks SS18 but includes the PBAF-specific subunits BAF45A and BRD7 [131,132]. The ncBAF (for non-canonical BAF complex) or GBAF is characterized by the incorporation of BRD9 and GLTSCR1/1L and lacks the cBAF subunits BAF47(SMARCB1), BAF57(SMARCE1), and BAF250 and the PBAF-specific subunits BAF200, BAF180, BAF45A (PHF10) and BRD7 [133–135].

The heterogeneity of these BAF complexes presumably leads to functional differences important in various developmental stages including pluripotency and cell lineage priming. In ESCs there are only three type of SWI/SNF complexes, esBAF — the embryonic version of the cBAF complex, GBAF and PBAF[136–138]. Highly abundant esBAF and GBAF complexes have primarily the Brg1 catalytic subunit as Brm is minimally expressed in naïve and primed stages of ESCs [136,139]. The embryonic stem (es)BAF complex is required for self-renewal and maintenance of ESCs, but its role in cell lineage priming and in the transition from naïve to primed ESCs has been unknown until a recent study[61]. The esBAF is essential for gene expression of many of the pluripotency TFs, as shown by esBAF binding to the key regulatory regions of the genes encoding for these factors and their diminished expression when Brg1 is deleted [139,140]. Mouse esBAF is distinguished by containing BAF250a not BAF250b, BAF60a/b instead of 60c and a homodimer of BAF155 (SMARCC1) instead of BAF155 and BAF170 heterodimer [131,139]. In human ESCs, there is a heterodimer of BAF170 and BAF155 which is important in pluripotency maintenance [141].

## BRG1 and esBAF role in pluripotency

The functional significance of Brg1 and the esBAF complex in mouse embryonic development is underscored by knockout of Brg1 causing lethality at the pre-implantation stage [142–144]. The embryonic BAF complex (esBAF) works cooperatively with the core pluripotency TFs (Oct4, Sox2, Nanog) [138,145]. Initially, Brg1 was known to bind promoters of pluripotency-related genes to regulate self-renewal [143]. Recently Oct4 has been shown to recruit Brg1 to cis-regulatory elements of pluripotency genes including Oct4 and is a self re-enforcing system of gene regulation [119]. While Brg1 is a linchpin component, other BAF complex subunits also wield significant influence in pluripotency preservation and differentiation.

Brm, although non-essential in mice, exhibits context-dependent effects [146,147]. Deleting Brm in ESCs does not compromise pluripotency but alters differentiation trajectory from precardiac mesoderm to non-mesodermal neural precursor lineage by preventing *de novo* accessibility at primed cardiac enhancers and activating neurogenic TF POU3F1 during cardiac differentiation [148]. BAF250A and BAF250B play unique roles in regulating pluripotency and lineage decisions [137,149]. Deleting BAF250A in ESCs results in reduced differentiation of cardiac mesodermal progenitors by leaving neuroectoderm lineage formation unaltered, illustrating its role in modulating lineage determinations by regulating OCT4 and β catenin recruitment to the lineage specific genes [150]. In addition to the well-delineated esBAF complex, the recently identified ncBAF assumes a crucial role in controlling the ESC transcriptome. Inhibiting the ncBAF subunit Brd9 triggers ESCs to adopt a morphology reminiscent of primed or epiblast ESCs, reduces colony-forming capacity, and down-regulates key pluripotency genes like Nanog and Klf4, underscoring Brd9's pivotal role in preserving the naive pluripotent state of ESCs [136]. Another esBAF component, Dpf2, influences meso-endodermal lineage differentiation by binding to and activating Tbx3 distal enhancer and modulating key factors like Tbx3 and Nanog, crucial for ESC self-renewal and differentiation [151–155]. BAF47, also part of the complex, controls ESC differentiation through Oct4 regulation, impacting the balance between pluripotency and differentiation [156]. Several other BAF subunits also influence pluripotency gene expression, highlighting the intricate regulatory network governing pluripotency and differentiation [138,145,149].

Beyond pluripotency maintenance, Brg1's functions extend to trophectoderm development, where it represses Nanog and Oct4 through an HDAC1 and Cdx2 mediated mechanism [157,158]. Moreover, significance of the BAF complex extends to the generation of induced pluripotent stem cells from differentiated cells. Brg1 is indispensable for reprogramming, and its overexpression bolsters reprogramming efficiency, while down-regulating Brm facilitates reprogramming [159–161]. Additionally, the inability of Brm or BAF170 to rescue the loss of Brg1 or BAF155, respectively, emphasizes the specificity and indispensable role of these subunits within the complex [138].

## The connection between Brg1 and its AT-hook in early development

**Figure 2.**
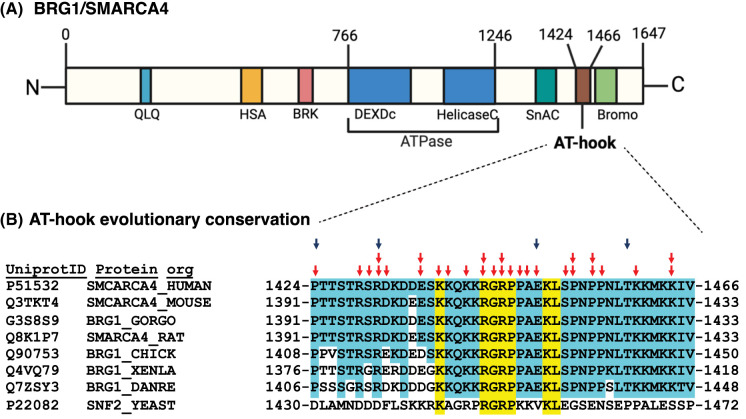
The AT-hook of BRG1 is evolutionarily conserved and targeted in various cancers and neurodevelopmental diseases. (**A**) Domain organization of the human SWI/SNF catalytic subunit, BRG1 shown including AT-hook motif at the C-terminus. Number across the top of the schematic represents amino acid positions. (**B**) Amino acid sequence alignment shown for the AT-hook motifs in human BRG1 and its homologs in Mus musculus (mouse), Gorilla gorilla gorilla (GORGO), Rattus norvegicus (RAT), Gallus gallus (CHICK), Xenopus laevis (XENLA), Danio rerio (DANRE), *Saccharomyces cerevisiae* (YEAST). Conserved residues are highlighted in yellow and blue. Red arrows indicate amino acids mutated in one or multiple cancer types reported in cBioPortal and blue arrows indicate amino acids mutated in neurodevelopmental diseases as reported in SPARK, DECIPHER, ClinVar [168].

The AT-hook motif, originally discovered in the High Mobility Group AT-hook 1 (HMGA1) protein, has emerged as a critical and conserved DNA-binding motif found in numerous chromatin-associated proteins, which at its core has the tripeptide R-G-R flanked by proline residues [162]. Crystallography and nuclear magnetic resonance investigations have shown the R-G-R inserts into the minor groove of DNA with flanking lysine residues in the AT-hook binding to the phosphate backbone of DNA [163–165]. Despite the extensive exploration of BRG1's helicase and bromodomain functions, the AT-hook domain has not been studied until recently and has gained attention for its distinct role in lineage commitment [61,166,167]. The AT-hook in the catalytic subunit of the SWI/SNF complex is part of a sub-category of AT-hooks called an extended AT-hook that is three times longer than other AT-hooks. The extended AT-hook has basic residues symmetrically extending 12–15 amino acids from the core AT-hook motif (Figure 2) [169,170]. The AT-hook in yeast SWI/SNF has been shown to have an auto-regulatory function that stimulates the nucleosome-dependent ATPase activity. Loss of the AT-hook does not perturb complex integrity, SWI/SNF's affinity for nucleosomes or the ability of SWI/SNF to be recruited through the acidic activation domain of Gal4-VP16 [61]. The AT-hook associates with another regulatory domain inside the catalytic subunit and the N-terminal tail of histone H3, suggesting potential ways in which it could positively regulate SWI/SNF activity.

The AT-hook in the mouse esBAF complex appears to have a similar role as evidenced by naïve-specific binding of pluripotency TFs being lost when the AT-hook of Brg1 is deleted as measured by ATAC-seq[61]. There is a similar loss of epiblast specific TF binding in the primed staged with Zic2, Zic3 and Six4. Binding of the pluripotency TFs to sites that are in both naïve and primed stages or to their own genes cis-regulatory regions are not disrupted by loss of the AT-hook, the later as seen by the levels of Oct4, Nanog and Sox2 protein not being affected by loss of the AT-hook[61]. Consequently, loss of the AT-hook does not affect self-renewal or pluripotency maintenance, unlike that observed when Brg1 is completely deleted [129,143]. Brg1 colocalizes with TFs bound to naïve- and primed-specific sites and these regions are likely active naïve- and primed-specific enhancers based on their genomic location and co-localization with H3K27ac and H3K4me1. Monomethylation of lysine 4 of histone 3 is thought to be an early step in activating enhancers and at these regions also requires the AT-hook of Brg1. Brg1 binding at these enhancers is not reduced by deletion of the AT-hook, which rules out the physical presence of Brg1 being crucial for TF binding or for H3K4me1 and instead points to the reduction in catalytic activity being the crucial factor. These findings indicate that the AT-hook positively regulating Brg1's remodeling activity is vital for the establishment of stage-specific enhancers.

Brg1 and its AT-hook is also important for cell lineage priming as seen after removing LIF and the two inhibitors used to maintain cells in the naïve stage and allowing cells to differentiate spontaneously[61]. Ectodermal markers Sox1 and Nestin, mesoderm marker Tbxt and the endoderm marker Sox17 are all down-regulated in AT-hook deleted cells, consistent with defects in cell lineage priming. The endoderm-specific marker Gata4 is aberrantly highly expressed when the AT-hook is deleted. These findings indicate the potential role of AT-hook motif of Brg1 in early neuronal and cardiac development. These observations are consistent with conserved residues in the AT-hook domain being mutated in several cancers and neurodevelopmental diseases (Figure 2).

Mapping changes in the sites of active transcription in the naïve and primed stages reveals there are large-scale changes in transcription in both stages when the AT-hook is deleted that shows Brg1 has role in both activating and repressing transcription[61]. There are genes encoding factors important in cell lineage priming that fail to be activated in the primed stage when the AT-hook is deleted. These proteins are involved in such process like signaling, organ and anatomical development, cell communication, cell differentiation and neural differentiation. There are another set of genes that are normally active in the naïve stage that are shutoff in the primed stage that fail to be switched off when the AT-hook is absent[61]. Interestingly, these genes encode many of the same factors shown in Figure 1 discussed that are necessary to establish the unique metabolic state characteristic of the naïve stage (Table 1). These factors are needed for high levels of oxidative phosphorylation, ATP generation and lipid metabolism. Some of these proteins are required to repress glycolysis that are subsequently down-regulated in the primed stage to make glycolysis dominant in the primed stage. Last of all, factors important for shortening the cell cycle and G-protein coupled signaling in the naïve stage are not repressed in the primed stage when the AT-hook of Brg1 is deleted. The failure of these factors to not be transcribed in the primed stage represents a failure to dismantle aspects of the pluripotent state important in switching from pluripotency to cell lineage priming.

## Conclusion

These studies for the first time indicate Brg1 has a critical role in cell lineage priming that is two-fold. One is to activate the necessary enhancers to transcribe the genes needed in the primed stage to make the factors needed for cell fate determination. The other surprising role of Brg1 is to repress expression of genes that encode metabolic, signaling and cell cycle factors that shape the pluripotent state, which need to be removed for cells to successfully exit pluripotency.

## Perspectives

- The SWI/SNF ATP-dependent chromatin remodeler plays pivotal roles in development from pluripotency to terminal differentiation, making it difficult to separate its distinct functions. Mutagenesis of SWI/SNF often leads to various diseases, the most notable being cancer and neurological disorders and reflects its role in development.
- To more fully understand the role of SWI/SNF, it is important to not only study the effects of completely deleting its catalytic subunit. A key example of this is the study of SWI/SNF in cell lineage priming, which was not possible by merely deleting Brg1 and instead required attenuating its activity by deleting an autoregulatory domain.
- In the future more work is needed to understand the domains involved in both regulating the activity of SWI/SNF as well as those that help target SWI/SNF to its appropriate genomic sites in a cell-type specific manner to better understand its molecular basis in development and diseases.

## Abbreviations

ESC: embryonic stem cell
ICM: inner cell mass
TF: transcription factor

## References

[1] Nichols, J. and Smith, A. (2009) Naive and primed pluripotent states. Cell Stem Cell 4, 487–492 10.1016/j.stem.2009.05.015 19497275

[2] Boroviak, T., Loos, R., Bertone, P., Smith, A. and Nichols, J. (2014) The ability of inner-cell-mass cells to self-renew as embryonic stem cells is acquired following epiblast specification. Nat. Cell Biol. 16, 516–528 10.1038/ncb2965 24859004 PMC4878656

[3] Smith, Z.D., Chan, M.M., Mikkelsen, T.S., Gu, H., Gnirke, A., Regev, A. et al. (2012) A unique regulatory phase of DNA methylation in the early mammalian embryo. Nature 484, 339–344 10.1038/nature10960 22456710 PMC3331945

[4] Nichols, J. and Smith, A. (2012) Pluripotency in the embryo and in culture. Cold Spring Harb. Perspect. Biol. 4, a008128 10.1101/cshperspect.a008128 22855723 PMC3405859

[5] Auclair, G., Guibert, S., Bender, A. and Weber, M. (2014) Ontogeny of CpG island methylation and specificity of DNMT3 methyltransferases during embryonic development in the mouse. Genome Biol. 15, 545 10.1186/s13059-014-0545-5 25476147 PMC4295324

[6] Bedzhov, I. and Zernicka-Goetz, M. (2014) Self-organizing properties of mouse pluripotent cells initiate morphogenesis upon implantation. Cell 156, 1032–1044 10.1016/j.cell.2014.01.023 24529478 PMC3991392

[7] Osorno, R. and Chambers, I. (2011) Transcription factor heterogeneity and epiblast pluripotency. Philos. Trans. R. Soc. Lond. B Biol. Sci. 366, 2230–2237 10.1098/rstb.2011.0043 21727128 PMC3130424

[8] Yeo, J.-C. and Ng, H.-H. (2013) The transcriptional regulation of pluripotency. Cell Res. 23, 20–32 10.1038/cr.2012.172 23229513 PMC3541660

[9] Weatherbee, B.A.T., Gantner, C.W., Iwamoto-Stohl, L.K., Daza, R.M., Hamazaki, N., Shendure, J. et al. (2023) Pluripotent stem cell-derived model of the post-implantation human embryo. Nature 622, 584–593 10.1038/s41586-023-06368-y 37369347 PMC10584688

[10] Weinberger, L., Ayyash, M., Novershtern, N. and Hanna, J.H. (2016) Dynamic stem cell states: naive to primed pluripotency in rodents and humans. Nat. Rev Mol. Cell Biol. 17, 155–169 10.1038/nrm.2015.28 26860365

[11] Thomson, J.A., Itskovitz-Eldor, J., Shapiro, S.S., Waknitz, M.A., Swiergiel, J.J., Marshall, V.S. et al. (1998) Embryonic stem cell lines derived from human blastocysts. Science 282, 1145–1147 10.1126/science.282.5391.1145 9804556

[12] Guo, G., von Meyenn, F., Santos, F., Chen, Y., Reik, W., Bertone, P. et al. (2016) Naive pluripotent stem cells derived directly from isolated cells of the human inner cell mass. Stem Cell Reports 6, 437–446 10.1016/j.stemcr.2016.02.005 26947977 PMC4834040

[13] Brons, I.G., Smithers, L.E., Trotter, M.W., Rugg-Gunn, P., Sun, B., Chuva de Sousa Lopes, S.M. et al. (2007) Derivation of pluripotent epiblast stem cells from mammalian embryos. Nature 448, 191–195 10.1038/nature05950 17597762

[14] Tesar, P.J., Chenoweth, J.G., Brook, F.A., Davies, T.J., Evans, E.P., Mack, D.L. et al. (2007) New cell lines from mouse epiblast share defining features with human embryonic stem cells. Nature 448, 196–199 10.1038/nature05972 17597760

[15] Gkountela, S., Zhang, K.X., Shafiq, T.A., Liao, W.W., Hargan-Calvopina, J., Chen, P.Y. et al. (2015) DNA demethylation dynamics in the human prenatal germline. Cell 161, 1425–1436 10.1016/j.cell.2015.05.012 26004067 PMC4458157

[16] Habibi, E., Brinkman, A.B., Arand, J., Kroeze, L.I., Kerstens, H.H., Matarese, F. et al. (2013) Whole-genome bisulfite sequencing of two distinct interconvertible DNA methylomes of mouse embryonic stem cells. Cell Stem Cell 13, 360–369 10.1016/j.stem.2013.06.002 23850244

[17] Ficz, G., Hore, T.A., Santos, F., Lee, H.J., Dean, W., Arand, J. et al. (2013) FGF signaling inhibition in ESCs drives rapid genome-wide demethylation to the epigenetic ground state of pluripotency. Cell Stem Cell 13, 351–359 10.1016/j.stem.2013.06.004 23850245 PMC3765959

[18] Leitch, H.G., McEwen, K.R., Turp, A., Encheva, V., Carroll, T., Grabole, N. et al. (2013) Naive pluripotency is associated with global DNA hypomethylation. Nat. Struct. Mol. Biol. 20, 311–316 10.1038/nsmb.2510 23416945 PMC3591483

[19] Hackett, J.A., Dietmann, S., Murakami, K., Down, T.A., Leitch, H.G. and Surani, M.A. (2013) Synergistic mechanisms of DNA demethylation during transition to ground-state pluripotency. Stem Cell Reports 1, 518–531 10.1016/j.stemcr.2013.11.010 24371807 PMC3871394

[20] Smith, Z.D., Chan, M.M., Humm, K.C., Karnik, R., Mekhoubad, S., Regev, A. et al. (2014) DNA methylation dynamics of the human preimplantation embryo. Nature 511, 611–615 10.1038/nature13581 25079558 PMC4178976

[21] Heard, E. (2004) Recent advances in X-chromosome inactivation. Curr. Opin. Cell Biol. 16, 247–255 10.1016/j.ceb.2004.03.005 15145348

[22] Kobayashi, S., Hosoi, Y., Shiura, H., Yamagata, K., Takahashi, S., Fujihara, Y. et al. (2016) Live imaging of X chromosome reactivation dynamics in early mouse development can discriminate naive from primed pluripotent stem cells. Development 143, 2958–2964 10.1242/dev.136739 27471261

[23] Okamoto, I., Patrat, C., Thepot, D., Peynot, N., Fauque, P., Daniel, N. et al. (2011) Eutherian mammals use diverse strategies to initiate X-chromosome inactivation during development. Nature 472, 370–374 10.1038/nature09872 21471966

[24] Nakamura, T., Okamoto, I., Sasaki, K., Yabuta, Y., Iwatani, C., Tsuchiya, H. et al. (2016) A developmental coordinate of pluripotency among mice, monkeys and humans. Nature 537, 57–62 10.1038/nature19096 27556940

[25] Marks, H., Kalkan, T., Menafra, R., Denissov, S., Jones, K., Hofemeister, H. et al. (2012) The transcriptional and epigenomic foundations of ground state pluripotency. Cell 149, 590–604 10.1016/j.cell.2012.03.026 22541430 PMC3398752

[26] Gafni, O., Weinberger, L., Mansour, A.A., Manor, Y.S., Chomsky, E., Ben-Yosef, D. et al. (2013) Derivation of novel human ground state naive pluripotent stem cells. Nature 504, 282–286 10.1038/nature12745 24172903

[27] Liu, X., Wang, C., Liu, W., Li, J., Li, C., Kou, X. et al. (2016) Distinct features of H3K4me3 and H3K27me3 chromatin domains in pre-implantation embryos. Nature 537, 558–562 10.1038/nature19362 27626379

[28] Yang, X., Hu, B., Hou, Y., Qiao, Y., Wang, R., Chen, Y. et al. (2018) Silencing of developmental genes by H3K27me3 and DNA methylation reflects the discrepant plasticity of embryonic and extraembryonic lineages. Cell Res. 28, 593–596 10.1038/s41422-018-0010-1 29463899 PMC5951795

[29] Zhang, W., Chen, Z., Yin, Q., Zhang, D., Racowsky, C. and Zhang, Y. (2019) Maternal-biased H3K27me3 correlates with paternal-specific gene expression in the human morula. Genes Dev. 33, 382–387 10.1101/gad.323105.118 30808660 PMC6446541

[30] Buecker, C., Srinivasan, R., Wu, Z., Calo, E., Acampora, D., Faial, T. et al. (2014) Reorganization of enhancer patterns in transition from naive to primed pluripotency. Cell Stem Cell 14, 838–853 10.1016/j.stem.2014.04.003 24905168 PMC4491504

[31] Yeom, Y.I., Fuhrmann, G., Ovitt, C.E., Brehm, A., Ohbo, K., Gross, M. et al. (1996) Germline regulatory element of Oct-4 specific for the totipotent cycle of embryonal cells. Development 122, 881–894 10.1242/dev.122.3.881 8631266

[32] Choi, H.W., Joo, J.Y., Hong, Y.J., Kim, J.S., Song, H., Lee, J.W. et al. (2016) Distinct enhancer activity of Oct4 in naive and primed mouse pluripotency. Stem Cell Reports 7, 911–926 10.1016/j.stemcr.2016.09.012 28157483 PMC5106531

[33] Karwacki-Neisius, V., Goke, J., Osorno, R., Halbritter, F., Ng, J.H., Weisse, A.Y. et al. (2013) Reduced Oct4 expression directs a robust pluripotent state with distinct signaling activity and increased enhancer occupancy by Oct4 and Nanog. Cell Stem Cell 12, 531–545 10.1016/j.stem.2013.04.023 23642364 PMC3650585

[34] Battle, S.L., Doni Jayavelu, N., Azad, R.N., Hesson, J., Ahmed, F.N., Overbey, E.G. et al. (2019) Enhancer chromatin and 3D genome architecture changes from naive to primed human embryonic stem cell states. Stem Cell Reports 12, 1129–1144 10.1016/j.stemcr.2019.04.004 31056477 PMC6524944

[35] Hawkins, R.D., Hon, G.C., Lee, L.K., Ngo, Q., Lister, R., Pelizzola, M. et al. (2010) Distinct epigenomic landscapes of pluripotent and lineage-committed human cells. Cell Stem Cell 6, 479–491 10.1016/j.stem.2010.03.018 20452322 PMC2867844

[36] Ahmed, K., Dehghani, H., Rugg-Gunn, P., Fussner, E., Rossant, J. and Bazett-Jones, D.P. (2010) Global chromatin architecture reflects pluripotency and lineage commitment in the early mouse embryo. PLoS One 5, e10531 10.1371/journal.pone.0010531 20479880 PMC2866533

[37] Liu, L., Leng, L., Liu, C., Lu, C., Yuan, Y., Wu, L. et al. (2019) An integrated chromatin accessibility and transcriptome landscape of human pre-implantation embryos. Nat. Commun. 10, 364 10.1038/s41467-018-08244-0 30664750 PMC6341076

[38] Yeh, C.Y., Huang, W.H., Chen, H.C. and Meir, Y.J. (2021) Capturing pluripotency and beyond. Cells 10, 3558 10.3390/cells10123558 34944066 PMC8700150

[39] Boroviak, T., Loos, R., Lombard, P., Okahara, J., Behr, R., Sasaki, E. et al. (2015) Lineage-specific profiling delineates the emergence and progression of naive pluripotency in mammalian embryogenesis. Dev. Cell 35, 366–382 10.1016/j.devcel.2015.10.011 26555056 PMC4643313

[40] Hackett, J.A. and Surani, M.A. (2014) Regulatory principles of pluripotency: from the ground state up. Cell Stem Cell 15, 416–430 10.1016/j.stem.2014.09.015 25280218

[41] Yan, L., Yang, M., Guo, H., Yang, L., Wu, J., Li, R. et al. (2013) Single-cell RNA-Seq profiling of human preimplantation embryos and embryonic stem cells. Nat. Struct. Mol. Biol. 20, 1131–1139 10.1038/nsmb.2660 23934149

[42] Nichols, J., Zevnik, B., Anastassiadis, K., Niwa, H., Klewe-Nebenius, D., Chambers, I. et al. (1998) Formation of pluripotent stem cells in the mammalian embryo depends on the POU transcription factor Oct4. Cell 95, 379–391 10.1016/S0092-8674(00)81769-9 9814708

[43] Blakeley, P., Fogarty, N.M., del Valle, I., Wamaitha, S.E., Hu, T.X., Elder, K. et al. (2015) Defining the three cell lineages of the human blastocyst by single-cell RNA-seq. Development 142, 3151–3165 10.1242/dev.131235 26293300 PMC4582176

[44] Orkin, S.H. and Hochedlinger, K. (2011) Chromatin connections to pluripotency and cellular reprogramming. Cell 145, 835–850 10.1016/j.cell.2011.05.019 21663790 PMC4858411

[45] Percharde, M., Lavial, F., Ng, J.H., Kumar, V., Tomaz, R.A., Martin, N. et al. (2012) Ncoa3 functions as an essential Esrrb coactivator to sustain embryonic stem cell self-renewal and reprogramming. Genes Dev. 26, 2286–2298 10.1101/gad.195545.112 23019124 PMC3475801

[46] Guo, G., Huss, M., Tong, G.Q., Wang, C., Li Sun, L., Clarke, N.D. et al. (2010) Resolution of cell fate decisions revealed by single-cell gene expression analysis from zygote to blastocyst. Dev. Cell 18, 675–685 10.1016/j.devcel.2010.02.012 20412781

[47] Adachi, K., Nikaido, I., Ohta, H., Ohtsuka, S., Ura, H., Kadota, M. et al. (2013) Context-dependent wiring of Sox2 regulatory networks for self-renewal of embryonic and trophoblast stem cells. Mol. Cell 52, 380–392 10.1016/j.molcel.2013.09.002 24120664

[48] Herchcovici Levy, S., Feldman Cohen, S., Arnon, L., Lahav, S., Awawdy, M., Alajem, A. et al. (2022) Esrrb is a cell-cycle-dependent associated factor balancing pluripotency and XEN differentiation. Stem Cell Reports 17, 1334–1350 10.1016/j.stemcr.2022.04.016 35594859 PMC9214067

[49] Faunes, F., Hayward, P., Descalzo, S.M., Chatterjee, S.S., Balayo, T., Trott, J. et al. (2013) A membrane-associated beta-catenin/Oct4 complex correlates with ground-state pluripotency in mouse embryonic stem cells. Development 140, 1171–1183 10.1242/dev.085654 23444350 PMC3585656

[50] Radice, G.L., Rayburn, H., Matsunami, H., Knudsen, K.A., Takeichi, M. and Hynes, R.O. (1997) Developmental defects in mouse embryos lacking N-cadherin. Dev. Biol. 181, 64–78 10.1006/dbio.1996.8443 9015265

[51] Collins, J.E. and Fleming, T.P. (1995) Epithelial differentiation in the mouse preimplantation embryo: making adhesive cell contacts for the first time. Trends Biochem. Sci. 20, 307–312 10.1016/S0968-0004(00)89057-X 7667889

[52] Bao, M., Cornwall-Scoones, J., Sanchez-Vasquez, E., Cox, A.L., Chen, D.Y., De Jonghe, J. et al. (2022) Stem cell-derived synthetic embryos self-assemble by exploiting cadherin codes and cortical tension. Nat. Cell Biol. 24, 1341–1349 10.1038/s41556-022-00984-y 36100738 PMC9481465

[53] Todaro, F., Campolo, F., Barrios, F., Pellegrini, M., Di Cesare, S., Tessarollo, L. et al. (2019) Regulation of kit expression in early mouse embryos and ES cells. Stem Cells 37, 332–344 10.1002/stem.2960 30566254 PMC8265211

[54] Yu, S., Zhou, C., He, J., Yao, Z., Huang, X., Rong, B. et al. (2022) BMP4 drives primed to naive transition through PGC-like state. Nat. Commun. 13, 2756 10.1038/s41467-022-30325-4 35589713 PMC9120449

[55] Evans, M.J. and Kaufman, M.H. (1981) Establishment in culture of pluripotential cells from mouse embryos. Nature 292, 154–156 10.1038/292154a0 7242681

[56] Manova, K., Nocka, K., Besmer, P. and Bachvarova, R.F. (1990) Gonadal expression of c-kit encoded at the W locus of the mouse. Development 110, 1057–1069 10.1242/dev.110.4.1057 1712701

[57] Tan, J., Zou, Y., Huang, Z.H., Zhang, Z.Q., Wu, L.P., Wu, X.W. et al. (2019) C-kit signaling promotes human pre-implantation 3PN embryonic development and blastocyst formation. Reprod. Biol. Endocrinol. 17, 75 10.1186/s12958-019-0521-8 31506068 PMC6737624

[58] Shakiba, N., White, C.A., Lipsitz, Y.Y., Yachie-Kinoshita, A., Tonge, P.D., Hussein, S.M.I. et al. (2015) CD24 tracks divergent pluripotent states in mouse and human cells. Nat. Commun. 6, 7329 10.1038/ncomms8329 26076835 PMC4490408

[59] Kagawa, H., Javali, A., Khoei, H.H., Sommer, T.M., Sestini, G., Novatchkova, M. et al. (2022) Human blastoids model blastocyst development and implantation. Nature 601, 600–605 10.1038/s41586-021-04267-8 34856602 PMC8791832

[60] Sperber, H., Mathieu, J., Wang, Y., Ferreccio, A., Hesson, J., Xu, Z. et al. (2015) The metabolome regulates the epigenetic landscape during naive-to-primed human embryonic stem cell transition. Nat. Cell Biol. 17, 1523–1535 10.1038/ncb3264 26571212 PMC4662931

[61] Saha, D., Hailu, S., Hada, A., Lee, J., Luo, J., Ranish, J.A. et al. (2023) The AT-hook is an evolutionarily conserved auto-regulatory domain of SWI/SNF required for cell lineage priming. Nat. Commun. 14, 4682 10.1038/s41467-023-40386-8 37542049 PMC10403523

[62] Takashima, Y., Guo, G., Loos, R., Nichols, J., Ficz, G., Krueger, F. et al. (2014) Resetting transcription factor control circuitry toward ground-state pluripotency in human. Cell 158, 1254–1269 10.1016/j.cell.2014.08.029 25215486 PMC4162745

[63] Birket, M.J., Orr, A.L., Gerencser, A.A., Madden, D.T., Vitelli, C., Swistowski, A. et al. (2011) A reduction in ATP demand and mitochondrial activity with neural differentiation of human embryonic stem cells. J. Cell Sci. 124, 348–358 10.1242/jcs.072272 21242311 PMC3021997

[64] Tsogtbaatar, E., Landin, C., Minter-Dykhouse, K. and Folmes, C.D.L. (2020) Energy metabolism regulates stem cell pluripotency. Front. Cell Dev. Biol. 8, 87 10.3389/fcell.2020.00087 32181250 PMC7059177

[65] Stincone, A., Prigione, A., Cramer, T., Wamelink, M.M., Campbell, K., Cheung, E. et al. (2015) The return of metabolism: biochemistry and physiology of the pentose phosphate pathway. Biol. Rev. Camb. Philos. Soc. 90, 927–963 10.1111/brv.12140 25243985 PMC4470864

[66] Leese, H.J. (2012) Metabolism of the preimplantation embryo: 40 years on. Reproduction 143, 417–427 10.1530/REP-11-0484 22408180

[67] Hardy, K., Hooper, M.A., Handyside, A.H., Rutherford, A.J., Winston, R.M. and Leese, H.J. (1989) Non-invasive measurement of glucose and pyruvate uptake by individual human oocytes and preimplantation embryos. Hum. Reprod. 4, 188–191 10.1093/oxfordjournals.humrep.a136869 2918073

[68] Cornacchia, D., Zhang, C., Zimmer, B., Chung, S.Y., Fan, Y., Soliman, M.A. et al. (2019) Lipid deprivation induces a stable, naive-to-primed intermediate state of pluripotency in human PSCs. Cell Stem Cell 25, 120–136.e10 10.1016/j.stem.2019.05.001 31155483 PMC7549840

[69] Tanosaki, S., Tohyama, S., Fujita, J., Someya, S., Hishiki, T., Matsuura, T. et al. (2020) Fatty acid synthesis is indispensable for survival of human pluripotent stem cells. iScience 23, 101535 10.1016/j.isci.2020.101535 33083764 PMC7509212

[70] Arena, R., Bisogno, S., Gasior, L., Rudnicka, J., Bernhardt, L., Haaf, T. et al. (2021) Lipid droplets in mammalian eggs are utilized during embryonic diapause. Proc. Natl Acad. Sci. U.S.A. 118, e2018362118 10.1073/pnas.2018362118 33649221 PMC7958255

[71] Zhao, T., Goh, K.J., Ng, H.H. and Vardy, L.A. (2012) A role for polyamine regulators in ESC self-renewal. Cell Cycle 11, 4517–4523 10.4161/cc.22772 23165208 PMC3562295

[72] James, C., Zhao, T.Y., Rahim, A., Saxena, P., Muthalif, N.A., Uemura, T. et al. (2018) MINDY1 is a downstream target of the polyamines and promotes embryonic stem cell self-renewal. Stem Cells 36, 1170–1178 10.1002/stem.2830 29644784

[73] Zhao, J., Yao, K., Yu, H., Zhang, L., Xu, Y., Chen, L. et al. (2021) Metabolic remodelling during early mouse embryo development. Nat. Metab. 3, 1372–1384 10.1038/s42255-021-00464-x 34650276

[74] Wang, J., Alexander, P., Wu, L., Hammer, R., Cleaver, O. and McKnight, S.L. (2009) Dependence of mouse embryonic stem cells on threonine catabolism. Science 325, 435–439 10.1126/science.1173288 19589965 PMC4373593

[75] Alexander, P.B., Wang, J. and McKnight, S.L. (2011) Targeted killing of a mammalian cell based upon its specialized metabolic state. Proc. Natl Acad. Sci. U.S.A. 108, 15828–15833 10.1073/pnas.1111312108 21896756 PMC3179072

[76] Shyh-Chang, N., Locasale, J.W., Lyssiotis, C.A., Zheng, Y., Teo, R.Y., Ratanasirintrawoot, S. et al. (2013) Influence of threonine metabolism on S-adenosylmethionine and histone methylation. Science 339, 222–226 10.1126/science.1226603 23118012 PMC3652341

[77] Bernstein, B.E., Mikkelsen, T.S., Xie, X., Kamal, M., Huebert, D.J., Cuff, J. et al. (2006) A bivalent chromatin structure marks key developmental genes in embryonic stem cells. Cell 125, 315–326 10.1016/j.cell.2006.02.041 16630819

[78] Carey, B.W., Finley, L.W., Cross, J.R., Allis, C.D. and Thompson, C.B. (2015) Intracellular alpha-ketoglutarate maintains the pluripotency of embryonic stem cells. Nature 518, 413–416 10.1038/nature13981 25487152 PMC4336218

[79] Nichols, J., Silva, J., Roode, M. and Smith, A. (2009) Suppression of Erk signalling promotes ground state pluripotency in the mouse embryo. Development 136, 3215–3222 10.1242/dev.038893 19710168 PMC2739140

[80] Simon, C.S., McCarthy, A., Woods, L., Staneva, D., Huang, Q., Linneberg-Agerholm, M. et al. Suppression of ERK signalling promotes pluripotent epiblast in the human blastocyst. bioRxiv 10.1101/2024.02.01.578414 PMC12304225 40721412

[81] Ogawa, K., Nishinakamura, R., Iwamatsu, Y., Shimosato, D. and Niwa, H. (2006) Synergistic action of Wnt and LIF in maintaining pluripotency of mouse ES cells. Biochem. Biophys. Res. Commun. 343, 159–166 10.1016/j.bbrc.2006.02.127 16530170

[82] Ying, Q.L., Wray, J., Nichols, J., Batlle-Morera, L., Doble, B., Woodgett, J. et al. (2008) The ground state of embryonic stem cell self-renewal. Nature 453, 519–523 10.1038/nature06968 18497825 PMC5328678

[83] Haegel, H., Larue, L., Ohsugi, M., Fedorov, L., Herrenknecht, K. and Kemler, R. (1995) Lack of beta-catenin affects mouse development at gastrulation. Development 121, 3529–3537 10.1242/dev.121.11.3529 8582267

[84] Dolatshad, N.F., Hellen, N., Jabbour, R.J., Harding, S.E. and Foldes, G. (2015) G-protein coupled receptor signaling in pluripotent stem cell-derived cardiovascular cells: implications for disease modeling. Front. Cell Dev. Biol. 3, 76 10.3389/fcell.2015.00076 26697426 PMC4673467

[85] Callihan, P., Mumaw, J., Machacek, D.W., Stice, S.L. and Hooks, S.B. (2011) Regulation of stem cell pluripotency and differentiation by G protein coupled receptors. Pharmacol. Ther. 129, 290–306 10.1016/j.pharmthera.2010.10.007 21073897

[86] ter Huurne, M., Chappell, J., Dalton, S. and Stunnenberg, H.G. (2017) Distinct cell-cycle control in two different states of mouse pluripotency. Cell Stem Cell 21, 449–455.e4 10.1016/j.stem.2017.09.004 28985526 PMC5658514

[87] Stead, E., White, J., Faast, R., Conn, S., Goldstone, S., Rathjen, J. et al. (2002) Pluripotent cell division cycles are driven by ectopic Cdk2, cyclin A/E and E2F activities. Oncogene 21, 8320–8333 10.1038/sj.onc.1206015 12447695

[88] Neganova, I., Zhang, X., Atkinson, S. and Lako, M. (2009) Expression and functional analysis of G1 to S regulatory components reveals an important role for CDK2 in cell cycle regulation in human embryonic stem cells. Oncogene 28, 20–30 10.1038/onc.2008.358 18806832

[89] Fu, H., Zhang, W., Li, N., Yang, J., Ye, X., Tian, C. et al. (2021) Elevated retrotransposon activity and genomic instability in primed pluripotent stem cells. Genome Biol. 22, 201 10.1186/s13059-021-02417-9 34243810 PMC8268579

[90] Hayashi, K., Ohta, H., Kurimoto, K., Aramaki, S. and Saitou, M. (2011) Reconstitution of the mouse germ cell specification pathway in culture by pluripotent stem cells. Cell 146, 519–532 10.1016/j.cell.2011.06.052 21820164

[91] Smith, A. (2017) Formative pluripotency: the executive phase in a developmental continuum. Development 144, 365–373 10.1242/dev.142679 28143843 PMC5430734

[92] Kinoshita, M., Barber, M., Mansfield, W., Cui, Y., Spindlow, D., Stirparo, G.G. et al. (2021) Capture of mouse and human stem cells with features of formative pluripotency. Cell Stem Cell 28, 2180 10.1016/j.stem.2021.11.002 34861148 PMC8657791

[93] Wang, X., Xiang, Y., Yu, Y., Wang, R., Zhang, Y., Xu, Q. et al. (2021) Formative pluripotent stem cells show features of epiblast cells poised for gastrulation. Cell Res. 31, 526–541 10.1038/s41422-021-00477-x 33608671 PMC8089102

[94] Diamante, L. and Martello, G. (2022) Metabolic regulation in pluripotent stem cells. Curr. Opin. Genet. Dev. 75, 101923 10.1016/j.gde.2022.101923 35691147

[95] Kalkan, T., Olova, N., Roode, M., Mulas, C., Lee, H.J., Nett, I. et al. (2017) Tracking the embryonic stem cell transition from ground state pluripotency. Development 144, 1221–1234 10.1242/dev.142711 28174249 PMC5399622

[96] Reid, M.A., Dai, Z. and Locasale, J.W. (2017) The impact of cellular metabolism on chromatin dynamics and epigenetics. Nat. Cell Biol. 19, 1298–1306 10.1038/ncb3629 29058720 PMC5886854

[97] Cha, Y., Kim, T., Jeon, J., Jang, Y., Kim, P.B., Lopes, C. et al. (2021) SIRT2 regulates mitochondrial dynamics and reprogramming via MEK1-ERK-DRP1 and AKT1-DRP1 axes. Cell Rep. 37, 110155 10.1016/j.celrep.2021.110155 34965411 PMC8780843

[98] Scalise, M., Pochini, L., Console, L., Losso, M.A. and Indiveri, C. (2018) The human SLC1A5 (ASCT2) amino acid transporter: from function to structure and role in cell biology. Front. Cell Dev. Biol. 6, 96 10.3389/fcell.2018.00096 30234109 PMC6131531

[99] Amiri, M., Conserva, F., Panayiotou, C., Karlsson, A. and Solaroli, N. (2013) The human adenylate kinase 9 is a nucleoside mono- and diphosphate kinase. Int. J. Biochem. Cell Biol. 45, 925–931 10.1016/j.biocel.2013.02.004 23416111

[100] Layden, B.T., Newman, M., Chen, F., Fisher, A. and Lowe, Jr, W.L. (2010) G protein coupled receptors in embryonic stem cells: a role for Gs-alpha signaling. PLoS One 5, e9105 10.1371/journal.pone.0009105 20161705 PMC2816999

[101] Lynch, J.R. and Wang, J.Y. (2016) G protein-coupled receptor signaling in stem cells and cancer. Int. J. Mol. Sci. 17, 707 10.3390/ijms17050707 27187360 PMC4881529

[102] ten Berge, D., Kurek, D., Blauwkamp, T., Koole, W., Maas, A., Eroglu, E. et al. (2011) Embryonic stem cells require Wnt proteins to prevent differentiation to epiblast stem cells. Nat. Cell Biol. 13, 1070–1075 10.1038/ncb2314 21841791 PMC4157727

[103] Xu, Z., Robitaille, A.M., Berndt, J.D., Davidson, K.C., Fischer, K.A., Mathieu, J. et al. (2016) Wnt/β-catenin signaling promotes self-renewal and inhibits the primed state transition in naive human embryonic stem cells. Proc. Natl Acad. Sci. U.S.A. 113, E6382–E6390 10.1073/pnas.1613849113 27698112 PMC5081574

[104] Boward, B., Wu, T. and Dalton, S. (2016) Concise review: control of cell fate through cell cycle and pluripotency networks. Stem Cells 34, 1427–1436 10.1002/stem.2345 26889666 PMC5201256

[105] Takahashi, S., Kobayashi, S. and Hiratani, I. (2018) Epigenetic differences between naive and primed pluripotent stem cells. Cell. Mol. Life Sci. 75, 1191–1203 10.1007/s00018-017-2703-x 29134247 PMC5843680

[106] Lee, H.J., Hore, T.A. and Reik, W. (2014) Reprogramming the methylome: erasing memory and creating diversity. Cell Stem Cell 14, 710–719 10.1016/j.stem.2014.05.008 24905162 PMC4051243

[107] Okashita, N., Kumaki, Y., Ebi, K., Nishi, M., Okamoto, Y., Nakayama, M. et al. (2014) PRDM14 promotes active DNA demethylation through the ten-eleven translocation (TET)-mediated base excision repair pathway in embryonic stem cells. Development 141, 269–280 10.1242/dev.099622 24335252

[108] Okashita, N., Suwa, Y., Nishimura, O., Sakashita, N., Kadota, M., Nagamatsu, G. et al. (2016) PRDM14 drives OCT3/4 recruitment via active demethylation in the transition from primed to naive pluripotency. Stem Cell Reports 7, 1072–1086 10.1016/j.stemcr.2016.10.007 27866876 PMC5161533

[109] Yamamoto, M., Suwa, Y., Sugiyama, K., Okashita, N., Kawaguchi, M., Tani, N. et al. (2020) The PRDM14-CtBP1/2-PRC2 complex regulates transcriptional repression during the transition from primed to naive pluripotency. J. Cell Sci. 133, jcs240176 10.1242/jcs.240176 32661086

[110] Wu, J., Huang, B., Chen, H., Yin, Q., Liu, Y., Xiang, Y. et al. (2016) The landscape of accessible chromatin in mammalian preimplantation embryos. Nature 534, 652–657 10.1038/nature18606 27309802

[111] Factor, D.C., Corradin, O., Zentner, G.E., Saiakhova, A., Song, L., Chenoweth, J.G. et al. (2014) Epigenomic comparison reveals activation of “seed” enhancers during transition from naive to primed pluripotency. Cell Stem Cell 14, 854–863 10.1016/j.stem.2014.05.005 24905169 PMC4149284

[112] Ji, X., Dadon, D.B., Powell, B.E., Fan, Z.P., Borges-Rivera, D., Shachar, S. et al. (2016) 3D chromosome regulatory landscape of human pluripotent cells. Cell Stem Cell 18, 262–275 10.1016/j.stem.2015.11.007 26686465 PMC4848748

[113] Novo, C.L., Javierre, B.M., Cairns, J., Segonds-Pichon, A., Wingett, S.W., Freire-Pritchett, P. et al. (2018) Long-range enhancer interactions are prevalent in mouse embryonic stem cells and are reorganized upon pluripotent state transition. Cell Rep. 22, 2615–2627 10.1016/j.celrep.2018.02.040 29514091 PMC5863031

[114] Zofall, M., Persinger, J., Kassabov, S.R. and Bartholomew, B. (2006) Chromatin remodeling by ISW2 and SWI/SNF requires DNA translocation inside the nucleosome. Nat. Struct. Mol. Biol. 13, 339–346 10.1038/nsmb1071 16518397

[115] Yang, X., Zaurin, R., Beato, M. and Peterson, C.L. (2007) Swi3p controls SWI/SNF assembly and ATP-dependent H2A-H2B displacement. Nat. Struct. Mol. Biol. 14, 540–547 10.1038/nsmb1238 17496903

[116] Owen-Hughes, T., Utley, R.T., Cote, J., Peterson, C.L. and Workman, J.L. (1996) Persistent site-specific remodeling of a nucleosome array by transient action of the SWI/SNF complex. Science 273, 513–516 10.1126/science.273.5274.513 8662543

[117] Brahma, S., Udugama, M.I., Kim, J., Hada, A., Bhardwaj, S.K., Hailu, S.G. et al. (2017) INO80 exchanges H2A.Z for H2A by translocating on DNA proximal to histone dimers. Nat. Commun. 8, 15616 10.1038/ncomms15616 28604691 PMC5472786

[118] Trotter, K.W. and Archer, T.K. (2008) The BRG1 transcriptional coregulator. Nucl. Recept. Signal. 6, e004 10.1621/nrs.06004 18301784 PMC2254329

[119] King, H.W. and Klose, R.J. (2017) The pioneer factor OCT4 requires the chromatin remodeller BRG1 to support gene regulatory element function in mouse embryonic stem cells. Elife 6, e22631 10.7554/eLife.22631 28287392 PMC5400504

[120] Yu, Y., Chen, Y., Kim, B., Wang, H., Zhao, C., He, X. et al. (2013) Olig2 targets chromatin remodelers to enhancers to initiate oligodendrocyte differentiation. Cell 152, 248–261 10.1016/j.cell.2012.12.006 23332759 PMC3553550

[121] Chatterjee, N., Sinha, D., Lemma-Dechassa, M., Tan, S., Shogren-Knaak, M.A. and Bartholomew, B. (2011) Histone H3 tail acetylation modulates ATP-dependent remodeling through multiple mechanisms. Nucleic Acids Res. 39, 8378–8391 10.1093/nar/gkr535 21749977 PMC3201869

[122] Bowman, G.D. and Poirier, M.G. (2015) Post-translational modifications of histones that influence nucleosome dynamics. Chem. Rev. 115, 2274–2295 10.1021/cr500350x 25424540 PMC4375056

[123] Swygert, S.G. and Peterson, C.L. (2014) Chromatin dynamics: interplay between remodeling enzymes and histone modifications. Biochim. Biophys. Acta 1839, 728–736 10.1016/j.bbagrm.2014.02.013 24583555 PMC4099280

[124] Kim, J.H., Saraf, A., Florens, L., Washburn, M. and Workman, J.L. (2010) Gcn5 regulates the dissociation of SWI/SNF from chromatin by acetylation of Swi2/Snf2. Genes Dev. 24, 2766–2771 10.1101/gad.1979710 21159817 PMC3003194

[125] Ho, P.J., Lloyd, S.M. and Bao, X. (2019) Unwinding chromatin at the right places: how BAF is targeted to specific genomic locations during development. Development 146, dev178780 10.1242/dev.178780 31570369 PMC6803370

[126] Alver, B.H., Kim, K.H., Lu, P., Wang, X., Manchester, H.E., Wang, W. et al. (2017) The SWI/SNF chromatin remodelling complex is required for maintenance of lineage specific enhancers. Nat. Commun. 8, 14648 10.1038/ncomms14648 28262751 PMC5343482

[127] Park, Y.-K., Lee, J.-E., Yan, Z., McKernan, K., O'Haren, T., Wang, W. et al. (2021) Interplay of BAF and MLL4 promotes cell type-specific enhancer activation. Nat. Commun. 12, 1630 10.1038/s41467-021-21893-y 33712604 PMC7955098

[128] Blümli, S., Wiechens, N., Wu, M.Y., Singh, V., Gierlinski, M., Schweikert, G. et al. (2021) Acute depletion of the ARID1A subunit of SWI/SNF complexes reveals distinct pathways for activation and repression of transcription. Cell Rep. 37, 109943 10.1016/j.celrep.2021.109943 34731603 PMC8578704

[129] Martin, B.J.E., Ablondi, E.F., Goglia, C., Mimoso, C.A., Espinel-Cabrera, P.R. and Adelman, K. (2023) Global identification of SWI/SNF targets reveals compensation by EP400. Cell 186, 5290–5307.e26 10.1016/j.cell.2023.10.006 37922899 PMC11307202

[130] Centore, R.C., Sandoval, G.J., Soares, L.M.M., Kadoch, C. and Chan, H.M. (2020) Mammalian SWI/SNF chromatin remodeling complexes: emerging mechanisms and therapeutic strategies. Trends Genet. 36, 936–950 10.1016/j.tig.2020.07.011 32873422

[131] Kaeser, M.D., Aslanian, A., Dong, M.Q., Yates, III, J.R. and Emerson, B.M. (2008) BRD7, a novel PBAF-specific SWI/SNF subunit, is required for target gene activation and repression in embryonic stem cells. J. Biol. Chem. 283, 32254–32263 10.1074/jbc.M806061200 18809673 PMC2583284

[132] Middeljans, E., Wan, X., Jansen, P.W., Sharma, V., Stunnenberg, H.G. and Logie, C. (2012) SS18 together with animal-specific factors defines human BAF-type SWI/SNF complexes. PLoS One 7, e33834 10.1371/journal.pone.0033834 22442726 PMC3307773

[133] Clapier, C.R., Iwasa, J., Cairns, B.R. and Peterson, C.L. (2017) Mechanisms of action and regulation of ATP-dependent chromatin-remodelling complexes. Nat. Rev. Mol. Cell Biol. 18, 407–422 10.1038/nrm.2017.26 28512350 PMC8127953

[134] Alpsoy, A. and Dykhuizen, E.C. (2018) Glioma tumor suppressor candidate region gene 1 (GLTSCR1) and its paralog GLTSCR1-like form SWI/SNF chromatin remodeling subcomplexes. J. Biol. Chem. 293, 3892–3903 10.1074/jbc.RA117.001065 29374058 PMC5858003

[135] Mashtalir, N., D'Avino, A.R., Michel, B.C., Luo, J., Pan, J., Otto, J.E. et al. (2018) Modular organization and assembly of SWI/SNF family chromatin remodeling complexes. Cell 175, 1272–1288.e20 10.1016/j.cell.2018.09.032 30343899 PMC6791824

[136] Gatchalian, J., Malik, S., Ho, J., Lee, D.S., Kelso, T.W.R., Shokhirev, M.N. et al. (2018) A non-canonical BRD9-containing BAF chromatin remodeling complex regulates naive pluripotency in mouse embryonic stem cells. Nat. Commun. 9, 5139 10.1038/s41467-018-07528-9 30510198 PMC6277444

[137] Yan, Z., Wang, Z., Sharova, L., Sharov, A.A., Ling, C., Piao, Y. et al. (2008) BAF250B-associated SWI/SNF chromatin-remodeling complex is required to maintain undifferentiated mouse embryonic stem cells. Stem Cells 26, 1155–1165 10.1634/stemcells.2007-0846 18323406 PMC2409195

[138] Ho, L., Ronan, J.L., Wu, J., Staahl, B.T., Chen, L., Kuo, A. et al. (2009) An embryonic stem cell chromatin remodeling complex, esBAF, is essential for embryonic stem cell self-renewal and pluripotency. Proc. Natl Acad. Sci. U.S.A. 106, 5181–5186 10.1073/pnas.0812889106 19279220 PMC2654396

[139] Ho, L., Jothi, R., Ronan, J.L., Cui, K., Zhao, K. and Crabtree, G.R. (2009) An embryonic stem cell chromatin remodeling complex, esBAF, is an essential component of the core pluripotency transcriptional network. Proc. Natl Acad. Sci. U.S.A. 106, 5187–5191 10.1073/pnas.0812888106 19279218 PMC2654397

[140] Ho, L., Miller, E.L., Ronan, J.L., Ho, W.Q., Jothi, R. and Crabtree, G.R. (2011) esBAF facilitates pluripotency by conditioning the genome for LIF/STAT3 signalling and by regulating polycomb function. Nat. Cell Biol. 13, 903–913 10.1038/ncb2285 21785422 PMC3155811

[141] Zhang, X., Li, B., Li, W., Ma, L., Zheng, D., Li, L. et al. (2014) Transcriptional repression by the BRG1-SWI/SNF complex affects the pluripotency of human embryonic stem cells. Stem Cell Reports 3, 460–474 10.1016/j.stemcr.2014.07.004 25241744 PMC4266000

[142] Bultman, S., Gebuhr, T., Yee, D., La Mantia, C., Nicholson, J., Gilliam, A. et al. (2000) A Brg1 null mutation in the mouse reveals functional differences among mammalian SWI/SNF complexes. Mol. Cell 6, 1287–1295 10.1016/S1097-2765(00)00127-1 11163203

[143] Kidder, B.L., Palmer, S. and Knott, J.G. (2009) SWI/SNF-Brg1 regulates self-renewal and occupies core pluripotency-related genes in embryonic stem cells. Stem Cells 27, 317–328 10.1634/stemcells.2008-0710 19056910

[144] Han, D., Jeon, S., Sohn, D.H., Lee, C., Ahn, S., Kim, W.K. et al. (2008) SRG3, a core component of mouse SWI/SNF complex, is essential for extra-embryonic vascular development. Dev. Biol. 315, 136–146 10.1016/j.ydbio.2007.12.024 18206867

[145] Ho, L. and Crabtree, G.R. (2010) Chromatin remodelling during development. Nature 463, 474–484 10.1038/nature08911 20110991 PMC3060774

[146] Reyes, J., Barra, J., Muchardt, C., Camus, A., Babinet, C. and Yaniv, M. (1998) Altered control of cellular proliferation in the absence of mammalian brahma (SNF2α). EMBO J. 17, 6979–6991 10.1093/emboj/17.23.6979 9843504 PMC1171046

[147] Thompson, K.W., Marquez, S.B., Lu, L. and Reisman, D. (2015) Induction of functional Brm protein from Brm knockout mice. Oncoscience 2, 349–361 10.18632/oncoscience.153 26097869 PMC4468321

[148] Hota, S.K., Rao, K.S., Blair, A.P., Khalilimeybodi, A., Hu, K.M., Thomas, R. et al. (2022) Brahma safeguards canalization of cardiac mesoderm differentiation. Nature 602, 129–134 10.1038/s41586-021-04336-y 35082446 PMC9196993

[149] Gao, X., Tate, P., Hu, P., Tjian, R., Skarnes, W.C. and Wang, Z. (2008) ES cell pluripotency and germ-layer formation require the SWI/SNF chromatin remodeling component BAF250a. Proc. Natl Acad. Sci. U.S.A. 105, 6656–6661 10.1073/pnas.0801802105 18448678 PMC2373334

[150] Lei, I., Tian, S., Chen, V., Zhao, Y. and Wang, Z. (2020) SWI/SNF component BAF250a coordinates OCT4 and WNT signaling pathway to control cardiac lineage differentiation. Front. Cell Dev. Biol. 7, 358 10.3389/fcell.2019.00358 32039194 PMC6987383

[151] Ivanova, N., Dobrin, R., Lu, R., Kotenko, I., Levorse, J., DeCoste, C. et al. (2006) Dissecting self-renewal in stem cells with RNA interference. Nature 442, 533–538 10.1038/nature04915 16767105

[152] Lu, R., Yang, A. and Jin, Y. (2011) Dual functions of T-box 3 (Tbx3) in the control of self-renewal and extraembryonic endoderm differentiation in mouse embryonic stem cells. J. Biol. Chem. 286, 8425–8436 10.1074/jbc.M110.202150 21189255 PMC3048727

[153] Weidgang, C.E., Russell, R., Tata, P.R., Kuhl, S.J., Illing, A., Muller, M. et al. (2014) TBX3 directs cell-fate decision toward mesendoderm. Stem Cell Reports 2, 747 10.1016/j.stemcr.2014.04.011 28081438 PMC4050486

[154] Waghray, A., Saiz, N., Jayaprakash, A.D., Freire, A.G., Papatsenko, D., Pereira, C.F. et al. (2015) Tbx3 controls Dppa3 levels and exit from pluripotency toward mesoderm. Stem Cell Reports 5, 97–110 10.1016/j.stemcr.2015.05.009 26095607 PMC4618439

[155] Zhang, W., Chronis, C., Chen, X., Zhang, H., Spalinskas, R., Pardo, M. et al. (2019) The BAF and PRC2 complex subunits Dpf2 and Eed antagonistically converge on Tbx3 to control ESC differentiation. Cell Stem Cell 24, 138–152.e8 10.1016/j.stem.2018.12.001 30609396 PMC6486830

[156] You, J.S., De Carvalho, D.D., Dai, C., Liu, M., Pandiyan, K., Zhou, X.J. et al. (2013) SNF5 is an essential executor of epigenetic regulation during differentiation. PLoS Genet. 9, e1003459 10.1371/journal.pgen.1003459 23637628 PMC3636213

[157] Carey, T.S., Cao, Z., Choi, I., Ganguly, A., Wilson, C.A., Paul, S. et al. (2015) BRG1 governs Nanog transcription in early mouse embryos and embryonic stem cells via antagonism of histone H3 lysine 9/14 acetylation. Mol. Cell. Biol. 35, 4158–4169 10.1128/MCB.00546-15 26416882 PMC4648823

[158] Wang, K., Sengupta, S., Magnani, L., Wilson, C.A., Henry, R.W. and Knott, J.G. (2010) Brg1 is required for Cdx2-mediated repression of Oct4 expression in mouse blastocysts. PLoS One 5, e10622 10.1371/journal.pone.0010622 20485553 PMC2868905

[159] Hansis, C., Barreto, G., Maltry, N. and Niehrs, C. (2004) Nuclear reprogramming of human somatic cells by xenopus egg extract requires BRG1. Curr. Biol. 14, 1475–1480 10.1016/j.cub.2004.08.031 15324664

[160] Singhal, N., Esch, D., Stehling, M. and Schöler, H.R. (2014) BRG1 is required to maintain pluripotency of murine embryonic stem cells. BioResearch Open Access 3, 1–8 10.1089/biores.2013.0047 24570840 PMC3929005

[161] Jiang, Z., Tang, Y., Zhao, X., Zhang, M., Donovan, D.M. and Tian, X. (2015) Knockdown of Brm and Baf170, components of chromatin remodeling complex, facilitates reprogramming of somatic cells. Stem Cells Dev. 24, 2328–2336 10.1089/scd.2015.0069 26121422 PMC4582692

[162] Reeves, R. and Nissen, M.S. (1990) The A.T-DNA-binding domain of mammalian high mobility group I chromosomal proteins. A novel peptide motif for recognizing DNA structure. J. Biol. Chem. 265, 8573–8582 10.1016/S0021-9258(19)38926-4 1692833

[163] Bewley, C.A., Gronenborn, A.M. and Clore, G.M. (1998) Minor groove-binding architectural proteins: structure, function, and DNA recognition. Annu. Rev. Biophys. Biomol. Struct. 27, 105–131 10.1146/annurev.biophys.27.1.105 9646864 PMC4781445

[164] Fonfría-Subirós, E., Acosta-Reyes, F., Saperas, N., Pous, J., Subirana, J.A. and Campos, J.L. (2012) Crystal structure of a complex of DNA with one AT-hook of HMGA1. PLoS One 7, e37120 10.1371/journal.pone.0037120 22615915 PMC3353895

[165] Huth, J.R., Bewley, C.A., Nissen, M.S., Evans, J.N.S., Reeves, R., Gronenborn, A.M. et al. (1997) The solution structure of an HMG-I(Y)–DNA complex defines a new architectural minor groove binding motif. Nat. Struct. Biol. 4, 657–665 10.1038/nsb0897-657 9253416

[166] Morrison, E.A., Sanchez, J.C., Ronan, J.L., Farrell, D.P., Varzavand, K., Johnson, J.K. et al. (2017) DNA binding drives the association of BRG1/hBRM bromodomains with nucleosomes. Nat. Commun. 8, 16080 10.1038/ncomms16080 28706277 PMC5519978

[167] Sanchez, J.C., Zhang, L., Evoli, S., Schnicker, N.J., Nunez-Hernandez, M., Yu, L. et al. (2020) The molecular basis of selective DNA binding by the BRG1 AT-hook and bromodomain. Biochim. Biophys. Acta Gene Regul. Mech. 1863, 194566 10.1016/j.bbagrm.2020.194566 32376391 PMC7350285

[168] Valencia, A.M., Sankar, A., van der Sluijs, P.J., Satterstrom, F.K., Fu, J., Talkowski, M.E. et al. (2023) Landscape of mSWI/SNF chromatin remodeling complex perturbations in neurodevelopmental disorders. Nat. Genet. 55, 1400–1412 10.1038/s41588-023-01451-6 37500730 PMC10412456

[169] Filarsky, M., Zillner, K., Araya, I., Villar-Garea, A., Merkl, R., Längst, G. et al. (2015) The extended AT-hook is a novel RNA binding motif. RNA Biol. 12, 864–876 10.1080/15476286.2015.1060394 26156556 PMC4615771

[170] Zillner, K., Filarsky, M., Rachow, K., Weinberger, M., Längst, G. and Nemeth, A. (2013) Large-scale organization of ribosomal DNA chromatin is regulated by Tip5. Nucleic Acids Res. 41, 5251–5262 10.1093/nar/gkt218 23580549 PMC3664807

